# Emotion recognition based on multimodal physiological electrical signals

**DOI:** 10.3389/fnins.2025.1512799

**Published:** 2025-03-05

**Authors:** Zhuozheng Wang, Yihan Wang

**Affiliations:** Faculty of Information Technology, Beijing University of Technology, Beijing, China

**Keywords:** emotion recognition, EEG signal, ECG signal, multimodal, deep learning

## Abstract

With the increasing severity of mental health problems, the application of emotion recognition techniques in mental health diagnosis and intervention has gradually received widespread attention. Accurate classification of emotional states is important for individual mental health management. This study proposes a multimodal emotion recognition method based on the fusion of electroencephalography (EEG) and electrocardiography (ECG) signals, aiming at the accurate classification of emotional states, especially for the three dimensions of emotions (potency, arousal, and sense of dominance). To this end, a composite neural network model (Att-1DCNN-GRU) is designed in this paper, which combines a one-dimensional convolutional neural network with an attention mechanism and gated recurrent units, and improves the emotion recognition by extracting the time-domain, frequency-domain, and nonlinear features of the EEG and ECG signals, and by employing a Random Forest approach to feature filtering, so as to improve the emotion recognition accuracy and robustness. The proposed model is validated on the DREAMER dataset, and the results show that the model achieves the three dimensions of emotion: value, arousal and dominance, with a high classification accuracy, especially on the ‘value’ dimension, with an accuracy of 95.95%. The fusion model significantly improves the recognition effect compared with the traditional emotion recognition methods using only EEG or ECG signals. In addition, to further validate the generalisation ability of the model, this study was also validated on the DEAP dataset, and the results showed that the model also performed well in terms of cross-dataset adaptation. Through a series of comparison and ablation experiments, this study demonstrates the advantages of multimodal signal fusion in emotion recognition and shows the great potential of deep learning methods in processing complex physiological signals. The experimental results show that the Att-1DCNN-GRU model exhibits strong capabilities in emotion recognition tasks, provides valuable technical support for emotion computing and mental health management, and has broad application prospects.

## 1 Introduction

In recent years, with the acceleration of the pace of life and the increase in social pressure, emotional problems have increasingly become an important factor affecting the physical and mental health of individuals and have had a far-reaching impact on economic and social development. Emotional state not only directly affects the mental health of individuals, but is also closely related to a variety of physiological diseases. The emotional dimensional model (VAD: Valence, Arousal, Dominance) provides a systematic framework for describing and analysing emotional states (Russell, 1980), so accurately identifying and classifying these emotional dimensions is of great theoretical and practical significance.

Most of the traditional emotion recognition methods rely on facial expressions, speech and text analysis, however, these methods are often affected by the individual’s subjective perception and environmental factors, making it difficult to accurately reflect the individual’s true emotional state. In contrast, physiological signals, especially electroencephalography (EEG) and electrocardiography (ECG), provide a more objective, real-time means of monitoring emotions. Over the past decade, a large number of neuropsychological studies have reported correlations between EEG signals and mood. There are two main regions of the brain associated with emotional activity: the amygdala (located in the anterior part of the temporal lobe, near the hippocampus) and the prefrontal cortex (covering part of the frontal lobe) (Alarcao and Fonseca, 2017). Moreover, with the continuous advancement of wearable device technology, it has become possible to acquire and analyse EEG and ECG signals in real time, providing new solutions for monitoring and managing emotional states. Therefore, this study proposes the fusion of EEG and ECG signals, combined with deep learning technology, to achieve accurate classification of the three dimensions of valence, arousal, and dominance in the emotion dimension model VAD, which has important theoretical value and application significance.

In recent years, emotion recognition methods based on physiological signals have been widely studied, and many scholars have proposed different emotion recognition models. For example, Picard et al. (2001) used the KNN method to classify eight emotions and achieved 81% classification accuracy. Huang et al. (2012) proposed a feature extraction algorithm called asymmetric spatial pattern (ASP), which solves the problems of high dimensionality and high noise of EEG signals, and achieves good results in emotional arousal and intensity detection with accuracies of 60% (VALUE) and 80% (AROUSAL). Atkinson and Campos (2016) combined a mutual information feature selection method and an SVM classifier to extend the emotion types and improve the accuracy of emotion classification of EEG signals, and the experimental results showed that the accuracy of this method was about 73% on the standard EEG dataset. In addition, Thammasan et al. (2016) investigated the application of deep confidence networks (DBNs) in music emotion recognition, combining fractal dimension (FD), power spectral density (PSD) and discrete wavelet transform (DWT) features for emotion classification, and experimental results showed that the accuracy of this method in emotion arousal classification reached 88.24 and 82.59%. In terms of ECG signals, Agrafioti et al. (2011) proposed an empirical modal decomposition (EMD)-based method to differentiate between different emotional modes through instantaneous frequency (Hilbert-Huang transform) and local oscillatory features, achieving a classification accuracy of 89%. Sarkar and Etemad (2020), on the other hand, proposed a self-supervised deepmulti-task learning framework to learn ECG representations through signal transformation recognition networks and applied it to emotion classification, which achieved more than 85% classification accuracy on multiple datasets, creating a new research advancement.

However, despite the good results of single EEG and ECG signals in emotion recognition, existing studies still face some limitations (Saganowski et al., 2022). Firstly, single signals often do not fully reflect emotional states; EEG has stronger signals in some emotional states, while ECG performs more significantly in other emotional states. Second, most of the existing methods are limited to single-modal signal analysis, neglecting the complementarity between multimodal signals. Finally, even with deep learning methods, how to effectively fuse EEG and ECG signals to improve classification accuracy and robustness is still an urgent problem.

To address the above challenges, this paper proposes an emotion recognition method based on the fusion of EEG and ECG signals, aiming to overcome the limitations in the existing methods through multimodal signal fusion and deep learning techniques. Compared with traditional emotion recognition methods, this paper innovatively combines deep learning with traditional signal processing techniques to advance the theoretical framework of emotion recognition by adaptively selecting features and fusing multimodal signals. This fusion approach enables emotion recognition not only to accurately capture subtle changes in emotions, but also to improve the robustness and adaptability of the system.

In recent years, many scholars have also adopted hybrid CNN and LSTM networks for EEG-based emotion recognition, and such methods improve the accuracy of emotion classification by extracting spatio-temporal features and capturing long time-dependent information (Chakravarthi et al., 2022). While in this paper, we combine CNN and GRU and introduce an attention mechanism (Att-1DCNN-GRU), which enables the model to automatically focus on the importance of different signals when processing multimodal signals, thus further optimising the emotion recognition effect. In addition, this paper validates the applicability of the model by validating it in different experimental environments and comparing it with data from other domains to ensure the consistency and broad applicability of the research results across multiple domains. Through interdisciplinary validation, we are able to ensure that the proposed method has strong generalisation capabilities in multiple application scenarios of emotion recognition. Finally, the experimental results show that the method in this paper achieves significant classification accuracy and better robustness compared to existing single-signal or traditional fusion methods in the classification task of the three emotion dimensions (valence, arousal, and dominance) in the emotion dimensionality model VAD, which validates the effectiveness of the proposed method.

## 2 Materials and methods

### 2.1 DREAMER dataset

The DREAMER dataset (Katsigiannis and Ramzan, 2017) is a multimodal physiological signal dataset specifically designed for emotion recognition research, aiming to identify and classify emotional states by analysing EEG and ECG signals. The DREAMER dataset stores EEG and ECG data before and after the 23 participants watched 18 movie clips, and scores of the three dimensions of Valence, Arousal and Dominance, respectively. Valence, Arousal, and Dominance.

The EEG data were collected by 14 electrodes covering different regions of the brain at a sampling rate of 128 Hz, which can reveal the electrical activity patterns of the brain in different emotional states; the ECG data were collected by a 2-channel ECG sensor at a sampling rate of 256 Hz, which provided detailed information on cardiac activity and helped to identify the physiological changes triggered by emotion. Participants rated their emotional experience using self-report after viewing each video. The rating dimensions included Valence, Arousal, and Dominance, each with a rating range of 1 to 5. These ratings provided an important reference for the training and validation of emotion recognition models, helping researchers to understand the relationship between physiological signals and subjective emotional experiences.

### 2.2 Signal preprocessing

In the emotion recognition task, the preprocessing of electroencephalogram (EEG) and electrocardiogram (ECG) signals is a key step in signal analysis, whose main purpose is to eliminate noise and pseudo-signals so as to improve the quality of the signals, and provide clearer and more reliable data for subsequent feature extraction and classification. Aiming at the characteristics of EEG and ECG signals, this paper adopts a variety of signal processing techniques to ensure the effectiveness and purity of the signals.

First, in order to effectively remove the industrial frequency interference, we use a 50 Hz trap filter. This filter is capable of accurate interference removal for the grid frequency (50 Hz), eliminating noise introduced by power equipment and the grid. By filtering out the 50 Hz frequency signal, the trap filter makes the low and high frequency portions of the EEG and ECG signals unaffected by industrial frequency interference.

Next, to further remove the low-frequency drift and high-frequency noise, a fourth-order Butterworth bandpass filter in the range of 0.5 to 45 Hz was used. The Butterworth filter is an important tool in signal processing because of its flat frequency response characteristics and distortion-free phase response. Its design ensures that the main frequency components of the EEG and ECG signals are preserved, while effectively filtering out low-frequency noise (e.g., myoelectric interference) and high-frequency noise (e.g., interference from electrical equipment). The bandpass filters are not only suitable for EEG and ECG signals, but are also widely used in audio processing, telecommunication and biomedical signal analysis due to their high fidelity and noise removal efficiency. The square function form of the amplitude of the Butterworth filter (Butterworth, 1930) is shown in Equation 1.


(1) $$A^{2}\left(\Omega \right)={\left|H_{a}\left(j\Omega \right)\right|}^{2}=\frac{1}{1+{\left(\frac{j\Omega }{j\Omega_{c}}\right)}^{2w}}$$

In order to remove the pseudo-signals introduced in the EEG signals due to eye movements (EOG), electromyography (EMG), etc., we used the technique of independent component analysis (ICA), which is a blind source separation technique that is widely used in the denoising of EEG signals (Hyvärinen and Oja, 2000). The basic principle of ICA is to break down the mixed signals into a number of statistically independent components, which represent the sources of the signals, through the demixing process. By applying ICA, we can extract pseudo-signals such as eye movements and EMG from EEG signals and retain the effective EEG activity components through denoising process. In practice, ICA can effectively separate the pseudo-signals that are not related to brain activities, thus improving the purity of EEG signals.

**Figure 1:**
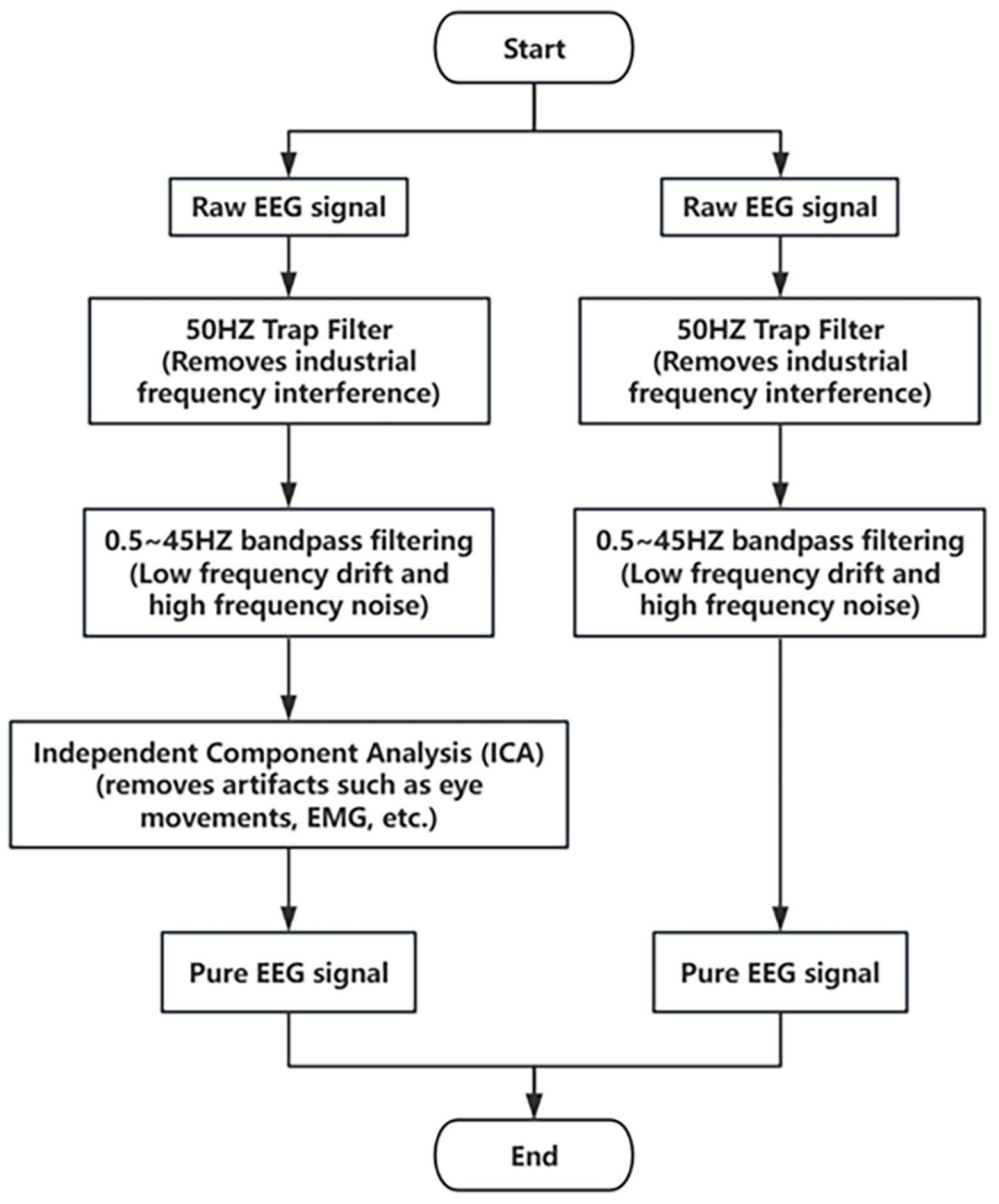
Preprocessing and denoising workflow for EEG and ECG signals.

After signal denoising, we slice the EEG and ECG signals to increase the number of samples and improve model training. Specifically, we slice each signal in units of 30 s to form multiple samples. Each EEG sample contains 3,840 data points (i.e., 30 s of data at a sampling rate of 128 Hz), and each ECG sample contains 7,680 data points (i.e., 30 s of data at a sampling rate of 256 Hz). Through the slicing operation, we not only increase the number of samples, but also are able to ensure that each signal fragment provides sufficient time-domain information for subsequent analyses while maintaining the signal time length and feature stability. The signal preprocessing flowchart used in this experiment is shown in Figure 1. These preprocessing steps ensure the quality of the EEG and ECG signals and provide clean signal data for subsequent feature extraction, model training and classification. By combining multiple signal processing techniques, this paper effectively removes noise and pseudo-signals, ensures the high quality of the data, and lays a solid foundation for the accuracy of the emotion recognition task.

### 2.3 Feature extraction and feature selection

In emotion recognition tasks, EEG and ECG signals contain rich physiological information that can reflect an individual’s emotional state. In order to extract effective emotional features from these signals, we perform time-domain, frequency-domain, and nonlinear analyses of EEG and ECG signals, respectively, from which we extract a variety of features. The time-domain features of EEG signals mainly include the maximum, minimum, mean, variance, peak-to-peak, kurtosis, and skewness, which effectively reflect the fluctuation of the signals and their statistical properties. The frequency domain features are then extracted by power spectral density (PSD) analysis, which is calculated for different frequency bands (Delta, Theta, Alpha, Beta, Gamma) to capture the energy distribution of the signal at different frequencies. Nonlinear features are then extracted by Sample Entropy (SE) and Detrended Fluctuation Analysis (DFA), which can reveal the complexity and nonlinear dynamic behaviour of the signal. These features can provide strong support for emotion recognition models, especially in the case of more subtle changes in the emotional state, where nonlinear features are particularly useful.

For feature extraction of ECG signals, we first identified R-wave locations in the ECG using an R-wave detection algorithm and then calculated RR intervals. Based on these RR intervals, heart rate variability (HRV) features were further extracted. The time-domain features of HRV include mean RR interval, heart rate, SDNN, RMSSD, NN50, and pNN50, which reflect the overall variability, short-term variability, and the statistical properties of the change between two heartbeats of heart rate, respectively. In addition, we analysed the short- and long-term variability of HRV by Poincaré plot features (SD1, SD2). Frequency domain features were then calculated by power spectral analysis, including low frequency (LF), high frequency (HF) and their ratio (LF/HF), which provide a quantitative analysis of sympathetic and parasympathetic activity. All these features are finally converged into a raw feature set containing both EEG and ECG signals.

However, these raw features contain a lot of redundant information, which may lead to overfitting during model training and increase the computational effort. Therefore, feature selection becomes an important step to improve the performance of emotion recognition models. In this study, the Random Forest (Random Forest) algorithm was used for feature selection. Random Forest is a powerful integrated learning method that can effectively reduce overfitting and improve the robustness of the model by constructing multiple decision trees and combining their results (Ho, 1998). In our experiments, we used 80 trees to train the Random Forest model, and filtered out the most discriminative features for the emotion recognition task by calculating the importance score of each feature. Eventually, after feature selection, nine most discriminative features were selected, and a detailed list of these features is shown in Table 1.

**Table 1:** Ranking of feature importance before and after random forest feature selection.

Rank	Feature name	Feature importance score
1	Mean RR interval	0.121
2	Heart Rate	0.115
3	Very-low-frequency power (VLF)	0.098
4	SD2	0.096
5	Standard Deviation (SDRR)	0.089
6	α-wave power spectral density	0.085
7	γ-wave power spectral density	0.082
8	β-wave power spectral density	0.079
9	DFA	0.077

This table shows the change in feature importance scores before and after random forest feature selection. In this table, it can be seen that after feature selection was performed, the most important features for the emotion classification task were selected. By calculating the importance score of each feature, we can see that these features play a decisive role in emotion recognition. The selected features include heart rate, RR interval, power spectral density in different frequency bands, sample entropy and DFA, which reflect the activity state of the heart and the brain and have strong emotion differentiation ability.

During the feature selection experiments, we also optimised the parameter settings of the Random Forest model and tried the effects of different numbers of decision trees on the effectiveness of feature selection. Specifically, we used settings of 50, 80 and 100 trees and compared the effects of these settings on model stability, computation time and accuracy. The experimental results show that the model achieves an optimal balance between feature selection stability and computational efficiency when the number of trees is 80. Fewer decision trees (e.g., 50) allowed for fast computation but were less stable and feature selection was not as effective as 80 trees, while increasing the number of trees (e.g., 100) improved stability but also significantly increased computation time. Therefore, 80 trees became the most suitable choice. Table 2 shows the experimental results for different numbers of decision trees.

**Table 2:** Random forest model parameter settings and experimental results.

Parameterisation	Feature selection stability	Computation time	Number of features finally selected	Training set accuracy	Test set accuracy
50	Instability	3.2 s	9	88.3%	85.6%
80	Stabilise	4.8 s	9	91.2%	88.9%
100	Stabilise	6.3 s	9	91.5%	89.1%

Through random forest feature selection, we are able to filter out the most discriminative features for the emotion recognition task from a large number of features, effectively reducing the feature dimensionality and improving the computational efficiency and performance of the model. The subset of sensitive features after feature selection (including 9 HRV features and 56 EEG signal features from EEG and ECG signals) provides efficient feature support for the subsequent emotion classification task. These selected features will be used for further emotion classification tasks in the subsequent training of emotion recognition models, leading to more accurate emotion state recognition.

With this feature selection method, we not only improved the computational efficiency of the model, but also enhanced the generalization ability and interpretability of the model. Eventually, the filtered feature set, consisting of 5 (number) × 2 (number of channels) = 10 (number of features) for ECG signals and 4 (number) × 14 (number of channels) = 56 (number of features) for EEG signals, was saved as a new MAT file, which provided a more streamlined and efficient data base for subsequent emotion recognition tasks.

### 2.4 Composite neural network design

#### 2.4.1 Network architecture design

**Figure 2:**
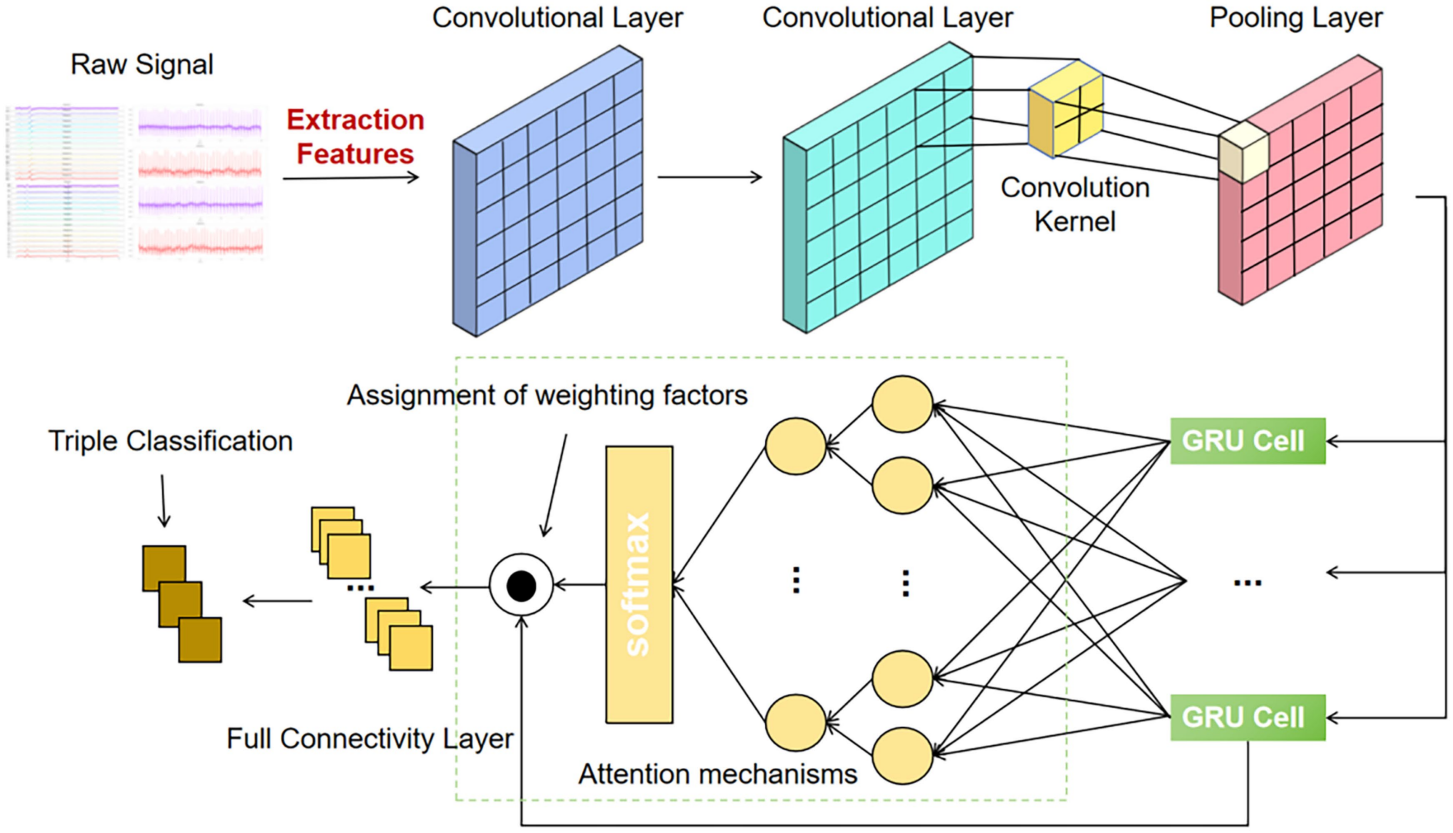
Architecture and detailed design of the hybrid neural network model (Att-1DCNN-GRU).

The composite neural network model proposed in this paper aims to effectively extract and process temporal features in emotion recognition tasks, and its specific design is shown in Figure 2. The model combines a one-dimensional convolutional neural network (1D CNN), a gated recurrent unit (GRU) and an attention mechanism. In the network design, the EEG and ECG signals after time domain, frequency domain and nonlinear feature extraction are firstly input, and these features are filtered and processed as inputs to the model. To extract the local features, the network first uses two convolutional layers, each with a number of 256 convolutional kernels and a convolutional kernel size of 3. Through these convolutional layers, the model is able to capture short-term local features in the input signals. In addition, the second convolutional layer is followed by a MaxPooling1D layer, an operation that not only effectively reduces the feature dimensions, but also prevents overfitting and improves the generalisation ability of the model.

After the convolutional layer, the network introduces a gated recurrent unit (GRU) layer to capture the temporal dependencies in the input signal. GRU, as a recurrent neural network (RNN) variant, is able to handle temporal data better, especially in emotion recognition tasks, and is able to learn long time dependencies. The output of the GRU layer is the sequential information of each timestep, which is further weighted in the subsequent attention mechanism is further weighted to highlight important features to improve the accuracy of emotion recognition. The attention mechanism, by assigning different weights to the features in the GRU output, enables the model to focus on those time steps that are more critical for emotion classification, thus enhancing the recognition of emotion-related features.

Next, the network further fuses the features from the GRU layer and the attention mechanism through the fully connected layer (Dense), and finally outputs the emotion classification results through the Softmax activation function. This output layer generates probability distributions of the three emotion dimensions (Valence, Arousal, and Dominance) for the classification of emotional states.

#### 2.4.2 Selection of optimizer and loss function

Optimizers for neural networks are used to update the weight parameters in a neural network to minimise the loss function of the neural network. Choosing the right optimizer can speed up training, improve the accuracy of the model and prevent overfitting. In this paper, 5,000 samples were randomly selected in the DREAMER dataset to apply the three current popular classifiers for comparison tests, and the Iteration parameters were adjusted according to the actual situation, and the specific experimental results are shown in Table 3. The results can be seen that Adam optimizer performs the best, the highest classification accuracy is 0.96 for the training set and 0.94 for the test set (epoch = 100). So in this study, Adam is chosen as the optimizer of the model, where the learning rate lr is set to 0.001. In this paper, the hidden layer of the hybrid network adopts one of the most used activation functions at present, i.e., the ReLU activation function (Xuejing et al., 2024). Because ReLU has a faster gradient drop during training, it can solve the problems of gradient vanishing and gradient explosion.

**Table 3:** Classification accuracy of different optimisers under different iteration.

Optimiser	Iteration = 20		Iteration = 50	
	Training set accuracy	Test set accuracy	Training set accuracy	Test set accuracy
**Adam**	0.85	0.87	**0.96**	**0.94**
Adagrad	0.71	0.72	0.83	0.81
RMSprop	0.81	0.76	0.90	0.88

The bold values in the table indicate the optimiser used in this paper and its corresponding accuracy.

In the study of emotion recognition problems, the categorical_cros-sentropy function is chosen as the loss function for the three-classification problem. categorical_crossentropy is one of the commonly used loss functions in multi-class classification problems, and it will compute the cross-entropy loss, which is used to evaluate the difference between the model prediction results and the real results, and update the model parameters by back propagation. The categorical cross entropy function is defined as shown in Equation 2.


(2) $$\text{loss}=-\frac{1}{m}\sum_{j=1}^{m}\sum_{i=1}^{m}y_{ji}\log{\hat{y}}_{ji}$$

Where denotes m number of samples, n denotes class, $y_{ji}$ denotes the true probability of class i, and ${\hat{y}}_{ji}$ denotes the predicted probability.

#### 2.4.3 Parameterization

In this paper, the grid search method (Krizhevsky et al., 2017) is used to tune and optimise the network parameters and hyperparameters and find the optimal combination of a set of parameters to be used as the parameters for model training. A 20% sample from the DREAMER dataset is randomly selected for testing. First, Iteration and Batchsize were set to 20 and 256, respectively. In the experiment, the number of filters and neurons was set to a multiple of 2 and the convolution kernel was set to 3 for tuning the network parameters, as shown in Table 4. Subsequently, after determining the network parameters, the selection of hyperparameters was carried out as shown in Table 5.

**Table 4:** Tuning of network parameters for the Att-1DCNN-GRU model.

Model	Conv_1	Conv_1	Kernel	GRU	Accuracy
M1	128	128	3	128	0.939
M2	128	128	3	256	0.946
M3	128	256	3	128	0.944
M4	128	256	3	256	0.952
M5	256	128	3	128	0.942
M6	256	128	3	256	0.949
M7	256	256	3	128	0.941
**M8**	**256**	**256**	**3**	**256**	**0.953**

The bold values shown in the table represent the parameter settings used in the model presented in this paper.

**Table 5:** Hyperparameter tuning and optimal settings for the Att-1DCNN-GRU model.

Model	Epoch	Batchsize	Accuracy
M1	50	128	0.871
**M2**	**50**	**256**	**0.966**
M3	80	128	0.911
M4	80	256	0.954
M5	100	128	0.913
M6	100	256	0.947

The bold values shown in the table represent the parameter settings used in the model presented in this paper.

As shown in Tables 4, 5, the final parameters of the model are: the number of filters in both convolutional layers is 256, the size of the convolutional kernel is 3, the Epoch is 50, and the Batchsize is 256.

### 2.5 Model algorithm design

In this paper, the softmax function is used to triple classify the output of the model. When dealing with multiclassification problems, the softmax activation function is usually used in the output layer to transform the output of the neural network into vectors representing the probabilities of the different classes. The mathematical expression of Softmax is shown in Equation 3.


(3) $$F\left(X_{i}\right)=\frac{\exp\left(X_{i}\right)\;i=0,1,2,\ldots ,k}{\sum_{j=0}^{k}\exp\left(X_{j}\right)}$$

Where $X_{i}$ is the input and $F\left(X_{i}\right)$ is the output. The numerator represents the probability to be found for each category and the denominator is the total probability. As can be seen from the formula, the calculated probabilities are in the range [0,1] and all probabilities sum to 1.

**Figure 3:**
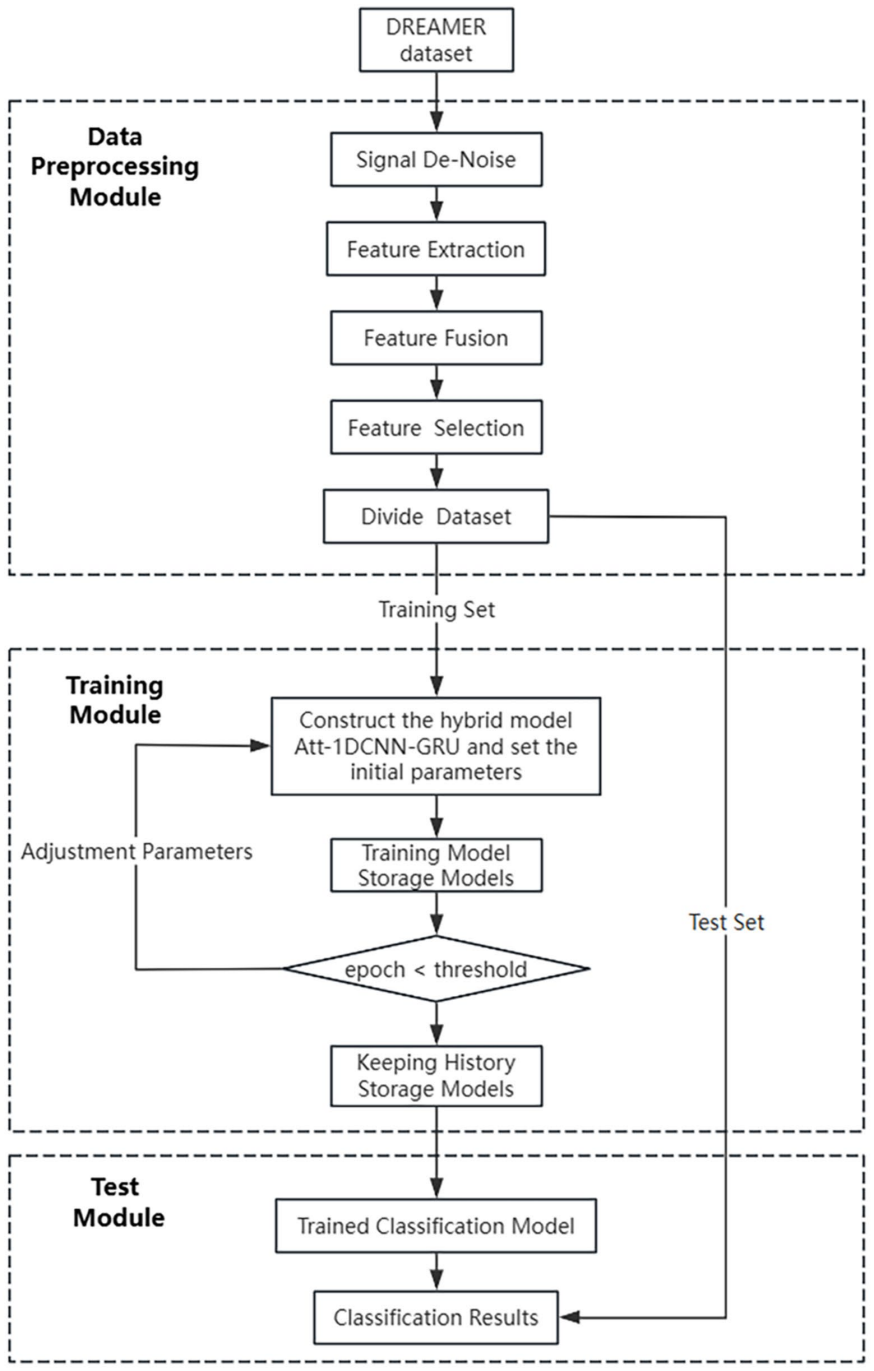
Overall flowchart of the Att-1DCNN-GRU algorithm for emotion recognition.

The algorithm flow of the model for emotion recognition is shown in Figure 3:

1. Extract the EEG and ECG data in the DREAME dataset, as well as the participants’ scores for the three items of VALENCE, AROUSAL, and DOMINANCE (because this paper designs a three-classification model, and the scores for these three items in the original dataset are 1 ~ 5, so in this paper, we will set those with scores of 1 and 2 to 0, those with scores of 3 to 1, and those with scores of 4 and 5 to 2), constituting the original data set.
2. Preprocess the EEG data and ECG data.
3. Extract the time-domain, frequency-domain, and nonlinear features of EEG and ECG and perform feature fusion, and use the method of random forest for feature selection.
4. The dataset is divided according to the ratio of 80% of the training set and 20% of the test set, and 10% of the training set is taken as the validation set, which is used to evaluate the performance of the model.
5. Train the hybrid network model with the training set, inversely update the weights and biases with the validation set, and save the trained model.
6. The test set is used to evaluate the effectiveness and accuracy of the algorithm. Classification for emotion recognition based on real labels and predicted labels.

## 3 Results

### 3.1 Evaluation indicators

The evaluation indicators selected for this paper are as follows:

(1) Accuracy, defined as the ratio of the number of correctly classified samples to the total number of samples, is calculated using the formula in Equation 4.

(4) $$\text{Accuracy}=\frac{\left|TP|+|TN\right|}{\left|TP|+|FP|+|TN|+|FN\right|}$$

(2) The precision rate, which is the ratio of the number of correctly categorised positive samples to the number of samples categorised as positive, measures the rate of checking accuracy, see Equation 5.

(5) $$\text{Precision}=\frac{\left|TP\right|}{\left|TP|+|FP\right|}$$

(3) Recall, which is the ratio of the number of correctly categorised positive samples to the number of actual positive samples, is measured as a check-perfect rate, see Equation 6.

(6) $$\text{Recall}=\frac{\left|TP\right|}{\left|TP|+|FN\right|}$$

(4) F1-score, a concept based on Precision and Recall, for which see Equation 7.

(7) $$F1-\text{score}=2*\frac{\text{Precision}*\text{Recall}}{\text{Precision}+\text{Recall}}$$

(5) Confusion matrix. The confusion matrix is also an effective model evaluation metric that provides a more intuitive visualisation of the classification accuracy in a data set. Confusion matrices are visualised in terms of probability values and sample sizes.

### 3.2 Experimental results

To ensure that the division between the training set, validation set and test set does not introduce any bias, this paper adopts a random segmentation method and pays special attention to the representativeness and balance of the dataset. In the specific operation, we randomly selected 80% of the samples from the DREAMER dataset as the training set, and the remaining 20% was used for the test set. Meanwhile, in order to avoid possible overfitting phenomenon, this paper also adopts the cross-validation technique in the training process. By dividing different data subsets several times and validating them, the distribution consistency of the training and test sets is ensured, and the impact of data bias on model performance is reduced. In addition, this paper also ensures that the proportion of emotional categories in each subset is as balanced as possible, thus ensuring that the distribution of emotional states in each subset is representative of the characteristics of the overall data. In order to further validate the validity and generalisation ability of the model, we plan to use more datasets for validation and testing in subsequent studies to enhance the credibility of the findings and to identify potential problems and improvement points.

**Figure 4:**
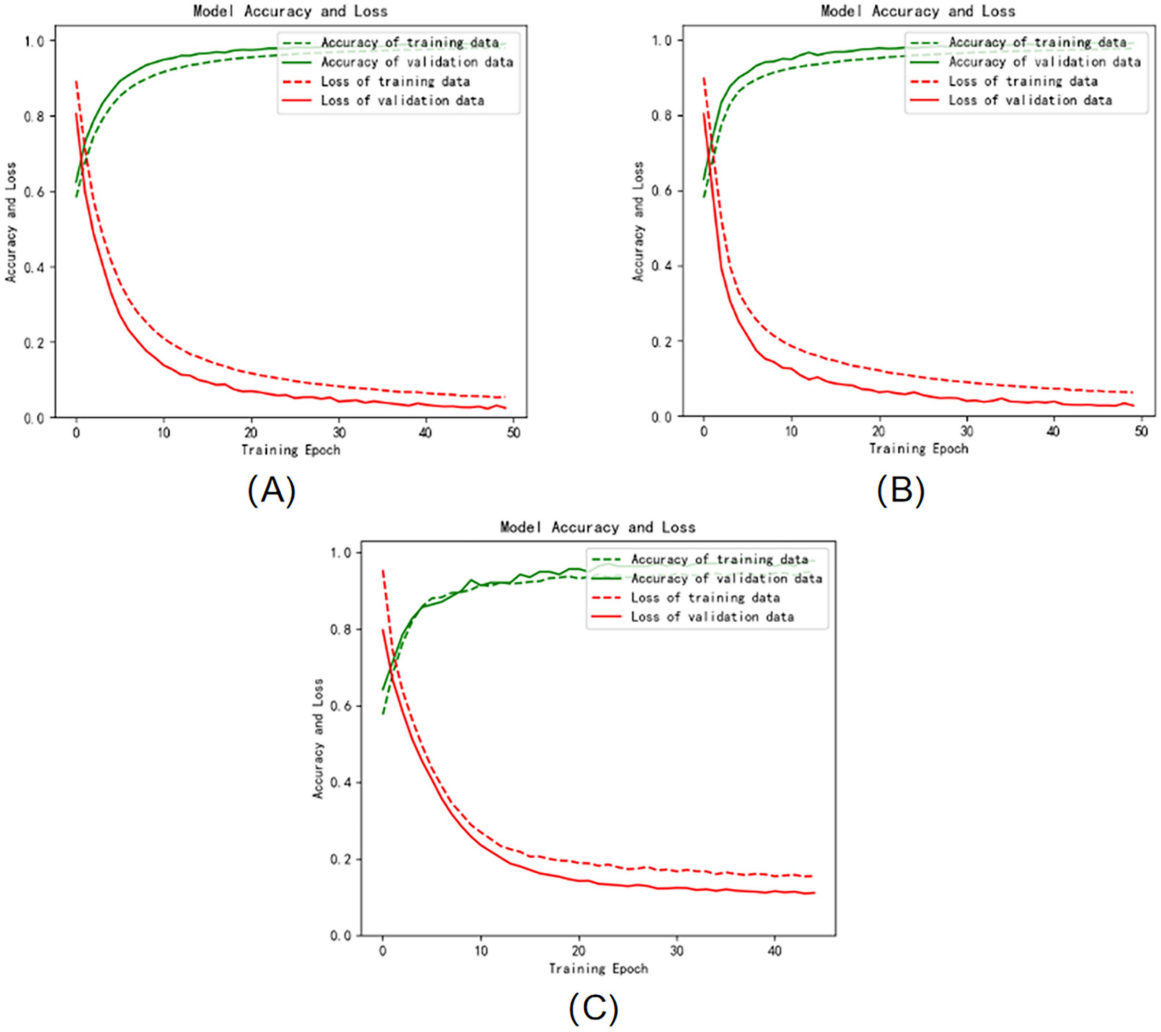
Training and validation accuracy and loss curves: **(A)** VALENCE dimension **(B)** AROUSAL dimension **(C)** DOMINANCE dimension.

The three graphs (A), (B), and (C) in Figure 4 show the iterative curves of the training process of the Att-1DCNN-GRU model proposed in this paper in the three dimensions of VALENCE, AROUSAL, and DOMINANCE, respectively, where the green dashed line represents the accuracy of the training data, the green solid line represents the accuracy of the validation data, and the red dashed line represents the loss of the training data. The red solid line represents the loss of the validation data. The training process of the model on the dataset are well behaved, convergence is fast, and no overfitting occurs. It is proved that the method proposed in this study can not only effectively perform emotion recognition, but also has high classification accuracy.

**Figure 5:**
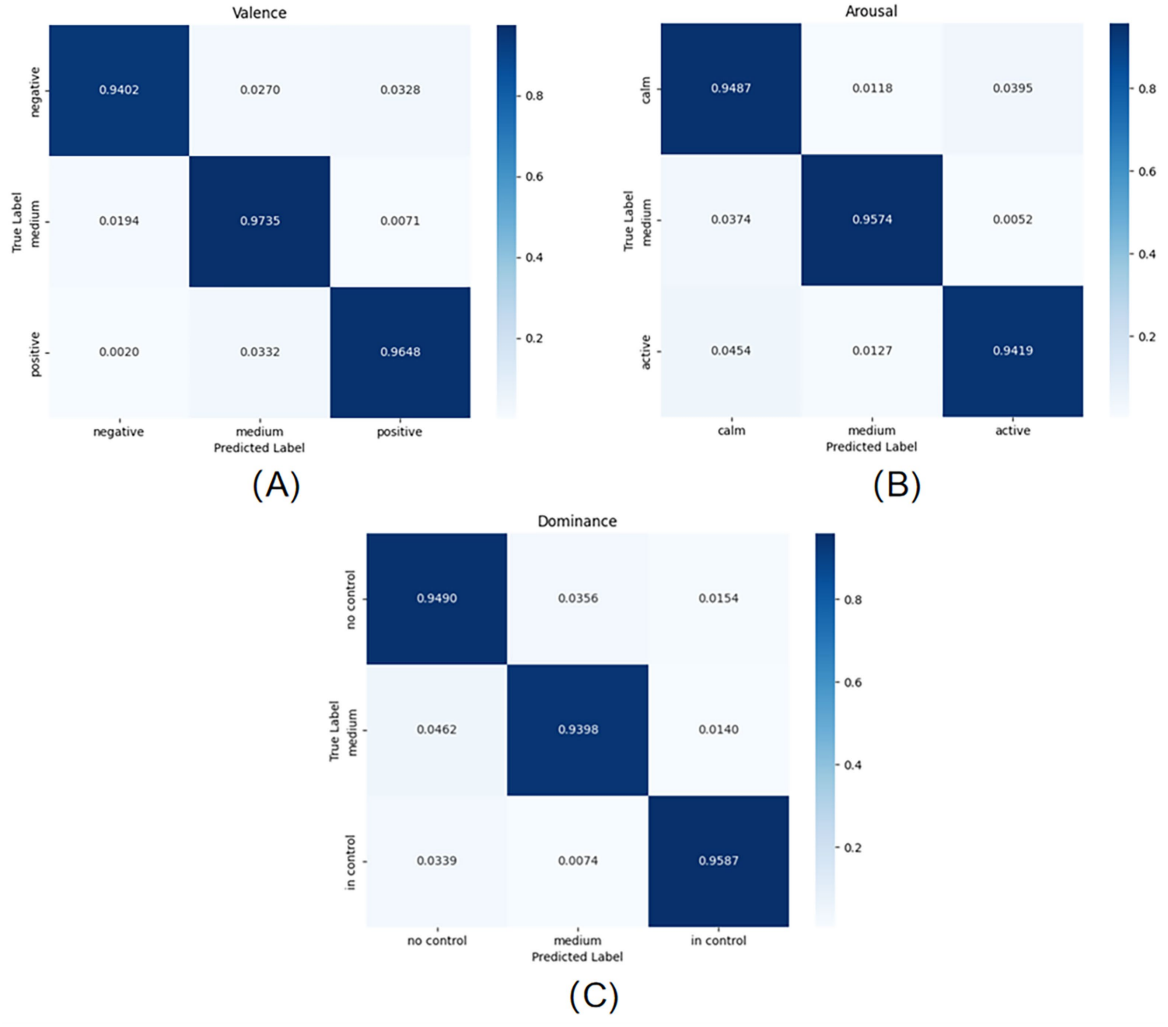
Confusion matrix for emotion classification on the test set: **(A)** VALENCE dimension **(B)** AROUSAL dimension **(C)** DOMINANCE dimension.

The three graphs (A), (B), and (C) in Figure 5 show the classification results of the model on the test set for the three scores of VALENCE, AROUSAL, and DOMINANCE, respectively, through the confusion matrix. As can be seen from Figure 5, the model has the best classification effect on VALENCE, which can reach 95.95%; followed by the classification effect on AROUSAL, which can reach 94.93%; and lastly, the classification effect on DOMINANCE, which can also reach 94.91%.

### 3.3 Comparative results and analysis of ablation experiments

In order to further evaluate the performance of the Att-1DCNN-GRU model proposed in this paper, this study conducted a multi-group comparison experiment on the emotion dimension VALENCE in the DREAMER dataset. The comparison models used include 1DCNN, GRU, 1DCNN-GRU, 1DCNN-Attention, GRU-Attention, and Att-1DCNN-GRU (the model proposed in this paper). The experimental results are shown in Table 6, indicating that the improved hybrid neural network model outperforms the other compared models in terms of prediction. The accuracy of the deep learning emotion recognition method is significantly higher than that of the traditional neural network algorithm, indicating that deep learning can adaptively extract valuable information from raw physiological data.

**Table 6:** Results of ablation experiments.

Model	Accuracy (VALENCE)	Accuracy (AROUSAL)	Accuracy (DOMINANCE)	Overall Accuracy
1DCNN	88.5%	87.3%	85.8%	87.2%
GRU	85.4%	84.6%	83.2%	84.4%
1DCNN-GRU	91.2%	90.5%	89.4%	90.4%
1DCNN-Attention	92.1%	91.7%	90.3%	91.3%
GRU-Attention	90.7%	89.4%	88.1%	89.4%
**Att-1DCNN-GRU**	**95.95%**	**94.93%**	**94.91%**	**95.26%**
EEG-only	87.2%	86.1%	84.9%	86.1%
ECG-only	83.6%	81.4%	80.7%	81.9%
DEAP Dataset	92.5%	91.3%	90.4%	92.5%

The bold values shown in the table represent the models presented in this paper and their corresponding accuracy rates.

In addition, it can be concluded from the ablation experiments that the improved hybrid model proposed in this paper has fast convergence, high accuracy, small loss and moderate training time, which proves that the model not only possesses high performance, but also provides theoretical support for the practical application of emotion recognition research. Especially in the training process, the model converges quickly and there is no overfitting phenomenon, which verifies the effectiveness of the method in the emotion recognition task.

In addition to the comparisons with other models, this study further conducted several additional comparison experiments to explore the impact of different data sources and datasets on model performance. Firstly, we conducted separate comparison experiments for the case of using EEG data alone and ECG data alone. The experimental results show that the accuracy of the model when using EEG data alone is significantly lower than the case of fusing EEG and ECG data, especially in the accuracy of recognising the emotion dimension. In contrast, although the use of ECG data alone achieved some success in some of the emotion dimensions, the recognition effect was far inferior to the fusion of the two due to the limitation of the ECG signal information.

To further validate the generality of the model, this study also tested it on the DEAP dataset, which is a typical emotion recognition dataset containing multimodal signals such as EEG and ECG. The experimental results show that the Att-1DCNN-GRU model achieves a classification accuracy of 92.5% on the DEAP dataset, which is a significant advantage over other traditional models. The results further demonstrate the generalisation ability of the model, showing that it not only achieves excellent results on the DREAMER dataset, but also adapts to other emotion recognition tasks, demonstrating strong adaptability.

In addition to this, we also compared with the latest model MS-MPHAN (Xuejing et al., 2024) published in 2024, which employs a multi-scale multi-channel hybrid attention mechanism and achieves an accuracy of 93.75% on the DEAP dataset. Although the accuracy of our model is slightly lower than the latest model by about 1%, we believe that by further optimising the feature extraction method, enhancing the fusion effect of spatio-temporal features, and adopting more advanced model architectures (e.g., self-supervised learning, graphical convolutional networks, etc.), we can overcome the current gap and improve the accuracy of the model to achieve a better performance in our future work.

## 4 Conclusion and future work

In this paper, we propose an emotion recognition method based on multimodal signal fusion, combining electroencephalogram (EEG) and electrocardiogram (ECG) signals, and by designing an improved composite neural network model Att-1DCNN-GRU, we successfully achieve accurate classification of the three dimensions of emotion (affect, arousal, and dominance). Through experimental verification, the model performs well in the emotion recognition task, especially the test results on the DEAP dataset, which proves that the fusion of EEG and ECG signals can effectively improve the accuracy and robustness of emotion recognition.

In the experimental process, the EEG and ECG signals were first rigorously pre-processed, including denoising, band-pass filtering, and other steps to ensure the purity and effectiveness of the signals. In terms of feature extraction, we adopted time-domain, frequency-domain and nonlinear methods to extract rich physiological features from EEG and ECG signals, and the most representative features were screened by the random forest method. In this way, the model is able to fully exploit the useful information in the signals and ensure a high classification performance.

The experimental results show that the Att-1DCNN-GRU model achieves a high level of classification accuracy in all three dimensions of emotion (VALENCE, AROUSAL, and DOMINANCE), with VALENCE having the highest classification accuracy of 95.95%. The fusion strategy of deep learning models demonstrates stronger classification ability and higher accuracy compared to traditional methods. In the comparison experiments, we also observed relatively low classification accuracy when using either EEG data or ECG data alone, further demonstrating the complementary nature of EEG and ECG signals and the advantages of multimodal fusion in emotion recognition.

In addition to the comparison of a single data source, we also included the validation of the DEAP dataset in our experiments to further extend the generalisation ability of the model. The experimental results show that the model performs with good stability and robustness on different datasets, providing strong evidence for the cross-dataset adaptability of emotion recognition techniques.

Although this study has achieved significant results, there are still some limitations that need to be addressed in future research. Firstly, despite the use of multimodal data fusion, the model is still sensitive to individual differences and the diversity of emotional states, and the adaptability of the model can be further improved in the future by introducing more personalised features and adaptive mechanisms. Second, the existing experiments mainly focus on the DEAP dataset and the DREAMER dataset, although these datasets are already representative, in order to enhance the credibility of the model, future research should consider validating the model using more publicly available datasets (e.g., AMIGOS, etc.) in order to test the model’s performance in different contexts.

In addition, this paper has made preliminary explorations on feature selection and model design, but the physiological significance of various types of features in the EEG and ECG signals and the specific relationship with the emotional state have not yet been explored in depth. Future studies can conduct more detailed studies on feature selection and fusion mechanisms through finer feature analysis, combined with psychological and physiological theories, in order to improve the interpretive and application value of the model.

In terms of model optimisation, more complex deep learning structures, such as dual-channel networks and temporal–spatial feature fusion networks, can be further explored in the future to improve the model’s processing capability on multimodal data. In addition, further optimisation of the attention mechanism and hierarchical structure design can also bring more flexibility and generalisation ability to the model.

Overall, this study provides a new idea and methodology in the field of emotion recognition, and makes significant progress in emotion recognition accuracy and robustness through multimodal signal fusion and innovative design of deep learning models. In the future, with the popularity of wearable devices and the increasing demand for mental health, emotion recognition technology is expected to become an important auxiliary diagnostic tool to provide personalised emotion calculation and mental health management solutions for individuals.

## Data Availability

Publicly available datasets were analyzed in this study. This data can be found at: https://zenodo.org/records/546113.
